# The redundancy of NMR restraints can be used to accelerate the unfolding behavior of an SH3 domain during molecular dynamics simulations

**DOI:** 10.1186/1472-6807-11-46

**Published:** 2011-11-24

**Authors:** Nathalie Duclert-Savatier, Leandro Martínez, Michael Nilges, Thérèse E Malliavin

**Affiliations:** 1 Institut Pasteur, CNRS URA 2185, Unité de Bioinformatique Structurale, 25-28 rue du Dr Roux, F-75724 Paris Cedex 15, France; 2 Instituto de Física de São Carlos, Universidade de São Paulo. Av. Trabalhador São-carlense 400, 13566-590 São Carlos, SP, Brasil

**Keywords:** NMR, protein folding, SH3 domain, molecular dynamics simulation, QUEEN, contact order: Gaussian Network Model

## Abstract

**1 Abstract:**

**Background**

The simulation of protein unfolding usually requires recording long molecular dynamics trajectories. The present work aims to figure out whether NMR restraints data can be used to probe protein conformations in order to accelerate the unfolding simulation. The SH3 domain of nephrocystine (nph SH3) was shown by NMR to be destabilized by point mutations, and was thus chosen to illustrate the proposed method.

**Results**

The NMR restraints observed on the WT nph SH3 domain were sorted from the least redundant to the most redundant ones. Protein NMR conformations were then calculated with: (i) the set **full **including all NMR restraints measured on nph SH3, (ii) the set **reduced **where the least redundant restraints with respect to the set **full **were removed, (iii) the sets **random **where randomly picked-up restraints were removed. From each set of conformations, we recorded series of 5-ns MD trajectories. The β barrel architecture of nph SH3 in the trajectories starting from sets (i) and (iii) appears to be stable. On the contrary, on trajectories based on the set (ii), a displacement of the hydrophobic core residues and a variation of the β barrel inner cavity profile were observed. The overall nph SH3 destabilization agrees with previous experimental and simulation observations made on other SH3 domains. The destabilizing effect of mutations was also found to be enhanced by the removal of the least redundant restraints.

**Conclusions**

We conclude that the NMR restraint redundancy is connected to the instability of the SH3 nph domain. This restraint redundancy generalizes the contact order parameter, which is calculated from the contact map of a folded protein and was shown in the literature to be correlated to the protein folding rate. The relationship between the NMR restraint redundancy and the protein folding is also reminiscent of the previous use of the Gaussian Network Model to predict protein folding parameters.

## 3 Background

The analysis of protein folding by computational methods faces the enormous confor-mational space to be explored, and usually requires simulation lengths of at least several hundreds of ns. Simplified models [1,2], higher simulation temperature [3] or implicit solvation [4-7] can accelerate the process, but induce a deformation of the free energy surface.

Here, we propose a method based on the analysis of the NMR experimental restraints, to enhance the structure instability observed during explicit solvent molecular dynamics (MD) simulations with an all-atom force field. Each NMR restraint is ranked according to its redundancy with respect to the other restraints. Calculation of NMR protein con-formations is then performed, based on a reduced set of restraints, from which the least redundant restraints are removed. These conformations are modified starting points for MD simulations having more chances to display structure instability. MD simulations are recorded in the absence of any distance restraints, as the different restraint sets are only used for the generation of the starting points.

**Figure 1:**
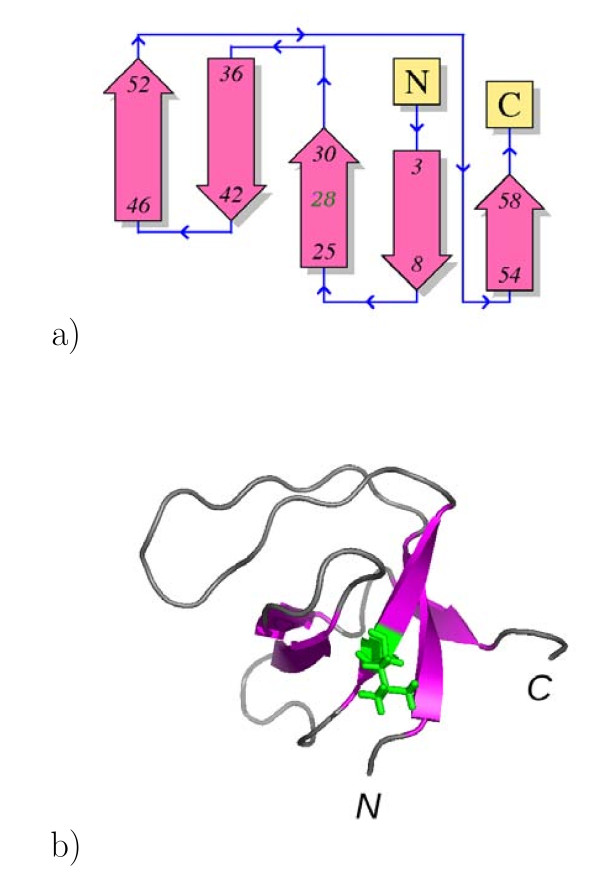
**Structure and topology of the SH3 domain** (a) Structure of the SH3 domain of the nephrocystine [9] (PDB entry: 1S1N) drawn in cartoon with β strands colored in magenta and the L28 colored in green and drawn in sticks. (b) Topology of the structure of the SH3 domain of the nephrocystine extracted from PDBsum [64]http://www.ebi.ac.uk/pdbsum/, with L28 written in green. The structure of the nph SH3 domain [9] reveals a classical β-barrel fold, surrounded by three loops: the RT loop (residues 8-23), the n-src loop (residues 30-37) and the distal hairpin (residues 41-47). This figure was produced with pymol 0.98 [10]

SH3 domain of nephrocystin (nph SH3), a β barrel protein associated with juvenile nephronophthisis [8], was used to illustrate this approach. Nph SH3 mutations associated to nephronophthisis, are located at the residue L28 in the strand β2 (Figure 1a). The structure of WT nph SH3 was determined by NMR [9] and the NMR analysis of the WT protein and of point mutants revealed that L28P is unfolded, whereas L28A is only partially folded. The impact of these mutations on the protein structure is quite intriguing, as residue L28 is pointing toward the solvent (Figure 1b). (This image was made with PyMOL software [10]).

The effect of removing the least redundant restraints was tested on WT nph SH3 by using three NMR restraints sets: (i) the set **full **including all measured NMR restraints, (ii) the set **reduced **where few least redundant restraints were removed from the set **full**, (iii) the sets **random**, where restraints were randomly removed from the set **full**. Three series of NMR conformations were calculated with these restraint sets, and display only small differences. Notably, the MD trajectories initiated from these different conformation series, display different trends. Similar β barrel shapes were observed during the trajectories starting from NMR conformations calculated with the sets **full **and **random**, whereas β barrel instability is found during trajectories started from the conformers calculated using the set **reduced**.

The effect of removing the least redundant restraints was also investigated on the nph SH3 mutants. The Leucine 28 sidechain was substituted by Proline or Alanine sidechains in the NMR conformers calculated using the restraint sets **full **and **reduced**. All MD trajectories started from these mutated conformations are less stable than the corresponding MD trajectories recorded on WT protein, but the instability is enhanced if starting points obtained with the set **reduced **are used.

The redundancy of each NMR restraint was evaluated through the calculation of the information it brings to the total NMR restraint set. The more information is brought by a restraint, the less redundant it is from the others. Conversely, low-information restraints depend a lot on the other restraints and are thus redundant. The information brought by each restraint was calculated using the approach QUEEN, introduced some years ago by Nabuurs et al [11].

The instability features observed on nph SH3 domain are in agreement with previous experimental and simulation information on the unfolding of SH3 domains, as the RT loop and the N and C terminal parts are the most destabilized regions. The changes in β barrel architecture are revealed by an original geometric analysis and are related to a packing defect of the hydrophobic core residues induced by the mutation of L28.

## 4 Methods

### 4.1 NMR conformers and restraints

The NMR restraints measured on the nph SH3 domain [9] were obtained from the PDB entry 1S1N. The approach QUEEN [11] was used to calculate the information brought by each restraint. From this information measure, the redundancy of each restraint with respect to the others was estimated. Three sets of restraints were then prepared: (i) the set **full**, including all restraints from 1S1N.mr, (ii) the set **reduced**, determined by discarding the nine least redundant restraints displaying the largest QUEEN information values, (iii) the set **random**, determined by discarding nine randomly chosen restraints, displaying low QUEEN information in the range 0-0.01.

These three sets of restraints were used to produce the corresponding sets (i), (ii) and (iii) of NMR conformations for the WT nph SH3 domain. NMR conformers were generated using CNS 1.1 [12]. The simulated annealing procedure of CNS was embedded into ARIA 2.2 [13]. A single iteration in geometric force field produced 1000 conformers, from which the 100 lowest-energy conformers were submitted to a water refinement step.

The NMR conformers were evaluated through: the coordinates RMSD between the backbone atoms, the number of violated distance restraints, the RMS of restraints violations [14], and the PROCHECK v3.5.4 [15] and WHATIF 5.1 [16] quality scores.

### 4.2 Molecular dynamics simulations

NMR conformers of the nph SH3 were starting points of the molecular dynamics (MD) simulations, performed with explicit solvent, and periodic boundary conditions at constant temperature (298 K) and pressure (1 atm). A cutoff distance of 10 Å was used to determine the water box size.

Trajectories were recorded using the package AMBER 9.0 [17] and the force field ff99SB [18]. A cutoff of 10 Å was used for Lennard-Jones interactions, and long-range electrostatic interactions were calculated with the Particle Mesh Ewald (PME) protocol [19]. The systems were neutralized using sodium counter-ions. Pressure was regulated with isotropic position scaling and a relaxation time of 1 ps, and temperature was regulated using a Langevin thermostat [20] with a collision frequency of 2 ps^-1 ^or a Berendsen thermostat [21] with a coupling time of 2 ps. The SHAKE algorithm [22] was used to keep rigid covalent bonds involving hydrogens, enabling a time step of 2 fs. Atom coordinates were saved at every ps.

Simulations were initiated by rounds of semi-restrained and unrestrained system minimization. The systems were thermalized to 298K for 20 ps at constant volume, while restraining the positions of the solute with a force constant of 25 kcal/(mol.Å^2^). The following equilibration protocol was then performed: 1 MD round of 5 ps at constant volume and 4 MD rounds of 2.5 ps were run while reducing the position restraints from 25 kcal/(mol.Å^2^) down to 5 kcal/(mol.Å^2^); eventually a last MD round of 70 ps was per-formed with a restraint of 2.5 kcal/(mol.Å^2^).

NMR conformers were selected from the conformers sets (i), (ii), (iii) described in subsection "NMR conformers and restraints", as starting points for MD simulations. For each conformer set, 8 runs of 5 ns MD trajectories were recorded varying: the thermo-stat controlling the system temperature (Berendsen or Langevin), the seed for the initial velocities generation (a = 71277, b = 22091) and the starting point chosen among the NMR conformer set. The mutations L28P and L28A were introduced in the WT NMR conformers calculated using the restraint sets **full **and **reduced**.

The Generalized Born Surface Area (GBSA) approach [23] was used to determine energy profiles per residues on the protein sequence. The generalized Born energy was calculated using the Onufriev method [24] and the following parameters: no counter-ions, a solvent dielectric constant of 80, and a protein dielectric constant of 1. The surface area was computed by recursively approximating a sphere around an atom, starting from an icosahedra [25] (gbsa = 1).

### 4.3 The β barrel architecture

An original geometric approach was used to analyze the SH3 β-barrel architecture (Additional file 1). First, the cylinder of maximum radius such that no protein backbone atom can be located inside (Additional file 1a) was determined to give the approximate main axis of the barrel. Second, a series of slabs (Additional file 1b) was defined along the main axis, and, within each slab, the maximum radius of the cylinder that can be fitted inside the volume defined by the protein backbone and the slab limits was calculated. As the slab width is 2 Å and the slab center is moved by steps of 0.5 Å, the cylinder radius obtained for each slab gives a local measure of the size of the inner β-barrel cavity, and the profile of the cylinder radius along the slabs thus provides an accurate and objective picture of the β barrel shape. As only the backbone atoms of the protein are included in the optimization and fitting procedures, the analysis focuses on the geometry of the β barrel, and considers the protein interior as a cavity.

The maximization of cylinder radius was implemented as a constrained smooth op-timization problem, and solved with the ALGENCAN solver [26,27]. Analytic derivatives were computed. Multiple initial points were considered to guarantee convergence to global solutions. This aspect is particularly relevant for the determination of the main axis, since, within each slab, the radius maximization is straightforward. The ALGEN-CAN solver is freely available at http://www.ime.usp.br/~egbirgin/tango and the package used in the present work is available at http://lm-utils.googlecode.com.

## 5 Theory

### 5.1 The restraint information

In a protein structure determination by NMR, the relationship between protein atomic coordinates and distance restraints can be described in the frame of the Distance Geometry approach [28]. The ensemble of protein atomic coordinates is considered as a 3D Euclidean object. Consequently, the upper and lower bounds of distance restraints verify the triangle inequalities, and the addition of a distance restraint potentially modifies the upper and lower bounds of all other restraints. As each restraint keeps the distance value into the lower/upper bound interval, it brings information improving the definition of the protein structure. Not all restraints bring the same quantity of information, as they do not have the same influence on the structure definition. In other words, low-information restraints are quite redundant and thus very dependent on the other restraints, whereas high-information restraints are much less redundant with respect to the other restraints defining the structure.

These properties of the NMR structure determination were known for a long time, but they were recently expressed in a quantitative way in the QUEEN approach [11], by proposing that the distance restraints defining a structure could be analyzed in the frame of information theory. The uncertainty *H*_*ij *_of the distance between spin nuclei *i *and *j*, can be expressed as:

(1) Hij=-∫ lijuij1uij-lij log1uij-lijdDij= log(uij-lij)

where *l*_*ij *_and *u*_*ij *_are respectively the lower and the upper bounds of the restraint.

The uncertainty of a given set *R *of restraints can be then expressed as:

(2) $$H_{structure|R}=\frac{1}{N}\overset{N}{\underset{i,j=1}{\sum }}H_{ij}$$

where *N *is the number of spin nuclei.

Therefore, the unique information *I*_*uni,r *_of a restraint *r*, applied between spins nuclei *i *and *j*, is defined as the difference of uncertainties between the sets *R *and *R*-1:

(3) $$I_{uni,r}=H_{structure|R-1}-H_{structure|R}$$

where *R*-1 represent the set of restraints others than *r *in the data-set, and *R *is the total set of restraints including *r*. The value *I*_*uni,r *_is different from the uncertainty *H*_*ij *_of the distance between spin nuclei *i *and *j*, as the addition of a restraint may induce a variation of all upper and lower bounds by application of the triangle inequalities.

The present work intends to use the QUEEN framework quantifying the redundancy of each restraint, in order to destabilize protein structure along its unfolding path.

### 5.2 Unique information and contact order

The unique information *I*_*uni,r *_of the QUEEN approach [11] can be related to the protein folding through the contact order parameter. The contact order *CO *[29] is defined from the ranges of the inter-residues contacts:

(4) $$CO=\frac{1}{N_{contact}N_{seq}}\underset{k,l}{\sum }|k-l|$$

where *k *and *l *are the residue numbers forming a native contact in the folded protein, *N*_*contact *_is the number of native contacts and *N*_*seq *_is the sequence size. The parameter *CO *is positive and smaller than 1. The parameter *CO *was shown [30,31] to be correlated to the folding rates, because of the decrease of conformational entropy associated with the formation of contacts [32].

To see how *CO *and *I*_*uni,r *_can be related, consider as a test case a folded protein, on which only the proximity information along the protein sequence was used to determine the upper and lower bound distances between residues. The constant upper limit for se-quential proximity being constant (*u*_*i,i*__+1 _= *u*) for all residues *i*, the upper *u*_*ij *_and lower *l*_*ij *_bounds between a given pair of residues *i *and *j*, would be given by:

(5) $$u_{ij}=u|i-j|$$

(6) $$l_{ij}=0$$

As the only sequential information is considered for the calculation of lower and upper bounds, no overall variation of the bounds is introduced when a restraint is added. Thus, the quantity of information *I*_*uni,r *_can be reduced to the uncertainty term *H*_*ij *_(Equation 1) of the distance between residues *i *and *j*. Using Equations 5, 6 and 1, *I*_*uni,r *_can be written as:

(7) $$I_{uni,r}=\log\left(u|i-j|\right)$$

The information *I*_*uni,r *_being related to the absolute difference value |*i *- *j*| also found in the *CO *definition, a new definition of the contact order could then be written as:

(8) $$CO*=\frac{1}{N_{contact}N_{seq}}\underset{r}{\sum }\exp\left(I_{uni,r}\right)∕u$$

In CO*, each term is proportional to the quantity of information brought by the corresponding restraint. By analogy to the definition of *CO *[32], one can thus hypothesize that *I*_*uni,r *_values are correlated with the protein folding rates and folding energy Removing the restraints of highest *I*_*uni,r *_during the NMR structure calculation will reduce CO* and thus increase the probability of unfolding of the conformations calculated with this reduced set of restraints. In the following, we will evaluate this tendency to instability by simulating MD trajectory starting from NMR conformations calculated using reduced restraints sets.

### 5.3 Unique information and folding cooperativity

As said previously, the information brought by a NMR restraint depends on the redundancy of this restraint with respect to the other restraints defining the protein structure. Redundant restraints correspond to highly cooperative contacts, which may be involved in the formation of the transition state ensemble (TSE) [33]. On the other hand, the least redundant restraints correspond to contacts with low cooperativity, which are formed after the appearance of TSE during the folding process. Reciprocally, when starting from the folded state, the removal of restraints displaying the largest *I*_*uni,r *_values should drive the protein conformation toward the TSE.

In the frame of the funnel model of protein folding [34], the reduction of conformational entropy from the funnel top to the bottom can be put in parallel with the increase of QUEEN information brought by each distance restraint, the contribution of information being equivalent to a reduction of entropy in information theory [35]. The removal of the most informative distance restraints induces an increase of conformational entropy, and thus a widening of the free-energy funnel, destabilizing the protein.

The NMR restraint set brings structural information, and, at a first sight, it seems paradoxical that such information can be related to protein unfolding, which depends on thermodynamics and kinetics parameters. Nevertheless, the lack of redundancy arises from the lack of NOE restraints in some protein regions, due to larger amplitude of the protein internal dynamics, and thus to the conformational entropy of the protein. NMR dynamical information was also proved in the past [36,37] to give information about the thermodynamic aspects of protein stability.

A relationship was already put in evidence some years ago [38] between the free energy changes related to hydrogen exchange, and the residue fluctuations predicted in the frame of the Gaussian Network Model (GNM) [39]. The previous observation of this effect gives a strong support to the model proposed here. Indeed, the elastic network describing the mechanics of a protein in GNM can be put in parallel with the set of NMR distance restraints. High redundancy of the NMR restraints involving the residue *i *corresponds in the frame of GNM to a large free energy contribution Δ*G*_*i *_of the residue *i *upon distortion of the protein coordinates. Following the observations of [39], the large free energy contributions Δ*G*_*i *_were shown to be correlated to the unfolding penalties revealed by hydrogen-exchange NMR experiments. The most redundant NMR restraints are thus the restraints whose disruption leads to the largest penalties for protein unfolding. Conversely, the disruption of the least redundant NMR restraints leads to lower unfolding penalties and is thus more prone to be disrupted early during the unfolding process.

The previous arguments are confirmed by the recent use [40] of the GNM to give information on protein unfolding pathways. In the work of Su et al [40], the contacts between protein residues, corresponding to the largest fluctuations calculated in the frame of GNM, are successively removed, and the corresponding variation of the protein contact map gives information about the possible protein unfolding pathways. The large similarity between the method proposed here and the protocol applied by [40], supports the use of NMR restraint redundancy to study protein unfolding.

## 6 Results

### 6.1 Effect of restraint redundancy on the NMR structure definition

The *I*_*uni,r *_values were calculated using QUEEN [11] on the 1S1N restraint list. The distribution of these values is dominated by nine long-range restraints (Additional file 2), distributed on different SH3 regions (Table 1): three restraints involve the β1 or β2 strand, two restraints involve the RT loop, the β3, β4 strand or the C terminal part, one restraint involves the distal hairpin or the n-src loop.

**Table 1:** The partners of the nine least redundant restraints sorted using QUEEN.

First restraint partner				Second restraint partner			
**Region**	**Residue**	**Number**	**Atom**	**Region**	**Residue**	**Number**	**Atom**

RT loop	Phe	11	Hδ#	β4	Pro	51	Hδ#
β1	Glu	4	Hγ#	C terminal	Glu	60	HN
n-src loop	Lys	33	HN	β3	Trp	38	Hα
β2	Leu	27	Hα	distal hairpin	Asp	42	Hα
β2	Leu	28	HN	β3	Lys	41	HN
RT loop	Ala	13	HN	RT loop	Thr	20	Hα
β1	Tyr	5	Hα	C terminal	Tyr	58	HN
β1	Tyr	5	Hβ#	β2	Val	29	Hγ2#
β3	Trp	38	HN	β4	Arg	52	HN

For each restraint partner, the protein region, the residue name and number and the hydrogen name are given. The hydrogens without stereospecific assignment are marked with a sign #.

Residues L27, L28 and V29 as well as residues A40, K41, D42 and E4, Y5 are in-volved in these nine restraints. Residue 28 is the mutated residue and L27, and V29 are its neighbors in the sequence, E4, Y5 and A40, K41, D42 being the neighboring residues in 3D space. The mutated residue L28, as well as its neighbors are involved in the least redundant restraints which were described in the section "Unique information and folding cooperativity" to define the last folding steps. It is thus not surprising that the perturbation of these residues by mutation, was shown by NMR [9] to have a large influence on the protein stability.

The relationship between the *I*_*uni,r *_values and the distribution of restraints in the protein structure was explored. Two restraint sets were used: (a) the 1S1N NMR restraints with lower and upper bounds set to 2.0 and 5.0 Å, (b) a synthetic set of restraints between all hydrogen pairs closer than 3 Å in the 1S1N PDB structure, with lower and upper bounds of 2 and 5 Å. In set (a), the nine restraints displaying the best *I*_*uni,r *_values includes five restraints already detected during the *I*_*uni,r *_calculation on the 1S1N restraint list. The redundancy of NMR restraints thus depends only partially on the distance bound values.

On the other hand, in the restraint set (b), all values *I*_*uni,r *_are null. Indeed, in the set (b) of restraints, for each couple of spins *k*, *j *for which a restraint *r *is considered, there are three other spins *i *and *l *and *m *which are connected to each other and connected to *i *and *j *by a restraint. This set of spins *i*, *j*, *k*, *l *and *m *form a rigid pentahedron. (Additional file 3a). If the connection between *k *and *j *is removed, the pentahedron is still rigid (Additional file 3b), and this implies that the upper and lower bounds of other connections are not changing. Thus, the information brought by the connection between *k *and *j *is equal to zero.

From the comparison of the two calculations run on sets (a) and (b), QUEEN thus seems to be the most sensitive to the restraints missing with respect to the full triangulation of the structure.

Three series of ARIA conformers were calculated using three sets of NMR restraints: (i) the set **full **including all 1S1N NMR restraints, (ii) the set **reduced **where the nine least redundant restraints described above were removed, (iii) the set **random **where nine randomly picked-up restraints were removed. The comparison of the ARIA conformers obtained from restraint sets **full **and **reduced **reveals that the removal of the nine least redundant restraints reduces slightly the convergence (Table 2), but the quality scores (Table 2) are similar for both conformers sets. Besides, the backbone mean RMSDs cross-calculated between the conformers obtained from both restraints sets, are of 2.3 ± 0.6 Å, only slightly larger than the RMSD values found among the two sets of con-formers (Table 2). Thus, the removal of least redundant restraints changes only slightly the obtained NMR structure. Unsurprisingly, the ARIA conformations obtained with the set **random **display features (data not shown) quite similar to the ones observed on the conformations calculated with the set **full**.

**Table 2:** Analysis of the 100 conformers of nph SH3 domain refined in water.

(a)	PROCHECK core (%)	PROCHECK allowed (%)	PROCHECK disallowed(%)	WHATIF Z score NQACHK
full	68.8 ± 6.2	27.4 ± 5.5	1.2 ± 1.5	-2.1 ± 0.7

reduced	64.5 ± 5.7	30.7 ± 5.2	1.9 ± 2.1	-2.5 ± 0.7

**(b)**	**WHATIF**	**WHATIF**	**WHATIF**	**WHATIF**
	**Z score**	**Z score**	**Z score**	
	**RAMCHK**	**C12CHK**	**BBCCHK**	**BMPCHK**

full	-5.3 ± 0.9	-2.7 ± 0.7	-3.9 ± 1.3	14.6 ± 3.8

reduced	-5.8 ± 0.8	-2.8 ± 0.6	-4.8 ± 1.5	15.0 ± 3.6

**(c)**	**Backbone RMSD (Å)**	**Heavy atom RMSD (Å)**	**NOE viol ≥ 0.3 Å**	**NOE RMS violations**

full	1.1 ± 0.2	1.8 ± 0.2	12.0 ± 3.6	1.1E-01 ± 2.2E-02

reduced	1.3 ± 0.3	2.1 ± 0.3	13.1 ± 3.6	1.23E-01 ± 2.8E-02

(a) PROCHECK: percentage of residues located in core region, allowed region, and dis-allowed region of the Ramachandran diagram. (a,b) The following WHATIF Z scores are given: 2nd generation packing quality (NQACHK), Ramachandran plot appearance (RAMCHK), the χ1/χ2 rotamer normality (C12CHK), backbone conformation (BBC-CHK) and the number of bumps (BMPCHK). (c) The convergence is analyzed using the coordinate RMSD value between the backbone and the heavy atoms, the fitting to the data is estimated from the number of restraint violations larger than 0.3 Å and the RMS of restraint violations.

The analysis of the redundancy of NMR restraints measured on the nph SH3 domains shows that the least redundant restraints involve the mutated residue as well as its neigh-bors. The least redundant restraints are also related to the lack of structure triangulation. Nevertheless, the removal of the least redundant restraints does not have large influence on the definition of the calculated protein structure.

### 6.2 The removal of the least redundant restraints induces β barrel instability during MD trajectories

**Figure 2:**
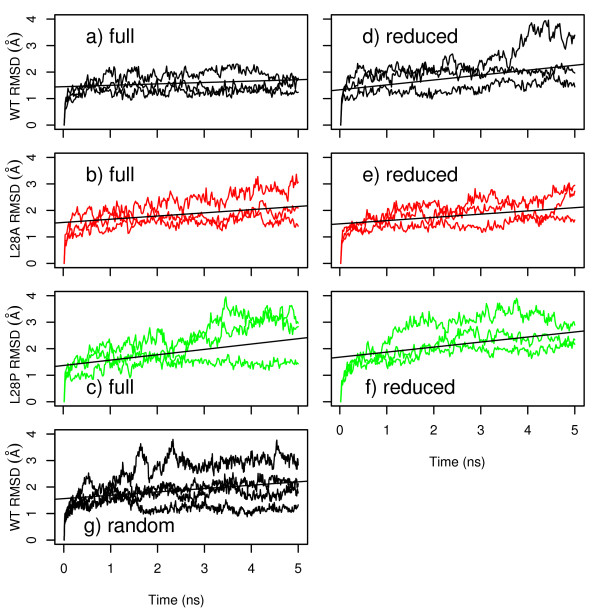
**Conformational drifts (Å) during 5 ns MD trajectories recorded on the WT protein (a,d,g), L28A mutant (b,e), and L28P mutant (c,f) of nph SH3 domain**. Only three curves are displayed, corresponding to the two extreme drifts and to a medium drift. The extreme conformational drifts observed among the MD runs are shown as curves, colored in black (WT), red (L28A) or green (L28P). The starting point of each trajectory was a WT nph SH3 conformer, generated using the sets **full **(a,b,c), **reduced **(d,e,f) and **random **(g) of NMR restraints. In the WT conformers, the L28 sidechain were then modified to A or P sidechains. When 8 runs were performed, the regression lines of the drifts are displayed as black lines. The drifts of the 4 runs performed for the randomly-reduced sets of restraints, are shown in the plot (g).

The conformational drift of the WT nph SH3 domain was compared during the MD simulations starting from NMR conformers generated using three NMR restraint sets: (i) **full **(Figure 2a), (ii) **reduced **(Figure 2d) and (iii) **random **(Figure 2g). The MD simulations based on the set **full **display coordinates RMSD in the range 1-2 Å (Figure 2a), whereas some of the runs based on the sets **reduced **(Figure 2d) and **random **(Figure 2g) display larger drifts up to 3 Å.

**Figure 3:**
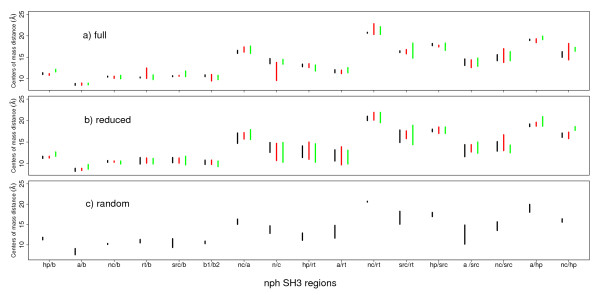
**Mean distances (Å) between structure centers of mass observed in MD simulations started from NMR conformers calculated using the three restraints sets: (a) the set full of NMR restraints of the PDB entry **1S1N, **(b) a set reduced of restraints where the restraints least redundant with respect to the full set were removed, (c) a set random of restraints, where randomly picked-up restraints were removed**. The segments describe the distance ranges, observed for WT (black), L28A (red) and L28P (green) sequences. The distances are calculated between centers of mass of several protein regions: hairpin loop (hp), α-helix (a), N/C terminal parts (nc, n, c), RT loop (rt), n-src loop (src), strands β1 (b1) and β2 (b2), written at the bottom of the plot.

The distances between centers of mass of the secondary structure elements of the nph SH3 domain were computed through MD simulations based on the sets **full**, **reduced **and **random **(Figure 3). For the sets **reduced **(Figure 3b, black segments) and **random **(Figure 3c, black segments), the distances increase with respect to the observations made for the set **full **(Figure 3a, black segments). These variations of protein architecture agree with the observations made on the nph SH3 conformational drift. The removal of NMR restraints, whatever they are randomly chosen or displaying the least redundancy, have a destabilizing effect on the SH3 structure.

Nevertheless, to complete this picture, structural parameters of the SH3 β barrel ge-ometry were analyzed. In the neighborhood of the residue 28, several distances were monitored between the residues sidechains involved in the SH3 hydrophobic core: Y5, L27, V29, W38, A40, V50 and L55. The mean distances between residues of the hy-drophobic core are similar whether MD simulations are based on the sets of restraints **full **or **random **(Additional files 4a and 4c, black bars). Thus, if randomly chosen restraints are removed, no change of the hydrophobic packing is observed near residue 28. On the other hand, if the set **reduced **is used (Additional file 4b, black bars), the distances V50/V29, L55/V29, Y5/V29, A40/L27 and V29/L27, increase.

**Figure 4:**
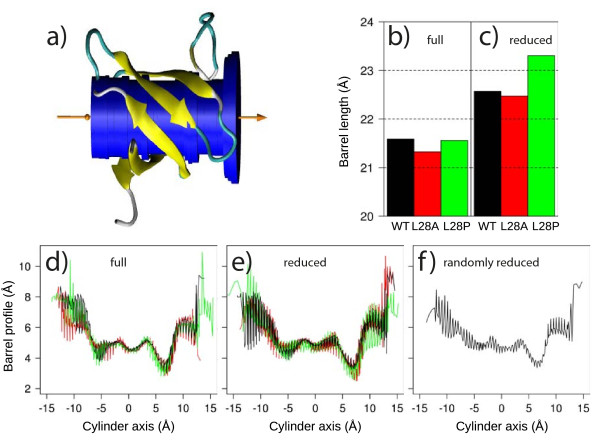
**Analysis of the β barrel shape**. (a) Example of a mean cylinder axis (orange) and of a set of cylinders (blue) describing the cavity interior of the SH3 β barrel. The SH3 structure is drawn in cartoon, with β strands colored in yellow. The cylinder axis origin is in the middle of cylinder. (b,c) Mean length of the β barrel obtained along MD simulations, as the position difference between the two extreme cylinders. The length was calculated for WT (black), L28A (red) and L28P (green) sequences, in simulations based on the sets **full **(b) and **reduced **(c) of NMR restraints. (d-f) Profiles of β barrel inner cavity described by cylinder radii plotted as interlaced values obtained from the runs. The corresponding MD simulations are based on the sets **full **(d), **reduced **(e) and **random **(f) of NMR restraints. The profiles are plotted along the coordinate of the slabs on the cylinder axis (Additional file 1), the axis origin being set at the middle of the cylinder axis drawn in plot (a).

Moreover, an original method was developed (see Methods and Additional file 1), to monitor the profile of the inner cavity of the β barrel (Figure 4a) (This image was made with VMD software [41]). The lengths of the β barrel are increased in the set **reduced **(Figure 4c) compared to the set **full **(Figure 4b). Similar profiles of the cylinder radii are observed (Figure 4d-f) for all simulations, in the range 3-5 Å, for the cylinder axis interval [-9, 9] Å and larger values in the range 5-10 Å, outside this interval. The barrel hoop oscillates: a minimum of 4.5 Å is observed at the origin, two maxima of about 5 Å are located around -2 and 3 Å, two minima smaller than 4 Å are located around -6 and 7 Å, the maxima located outside the cylinder axis interval [-9, 9] Å being less well defined. The maxima observed for the radii profile arise from residues of different protein regions: n-src loop and C terminal part (maximum position: -10 Å), n-src loop and α helix (maximum position: -5 Å), RT loop and distal hairpin (maximum positions: -2, 3 and 6-10 Å). The relative positions of the valves formed by n-src loop/C terminal part, n-src loop/α helix and RT loop/distal hairpin are thus essential to define the profile of the β barrel inner cavity.

The inner cavity profiles are plotted as black curves for the MD simulations of the WT nph SH3 domain (Figures 4d-f). One curve is obtained for each simulation by interlacing the values computed on each run. The profiles observed for the simulations based on the restraint sets **full **(Figure 4d) and **random **(Figure 4f), have smaller amplitudes than the profile computed from the simulations based on the set **reduced **(Figure 4e). The β barrel is thus more unstable if the least redundant restraints are removed than in the case of full or randomly-reduced restraint sets.

The simulations starting from NMR conformations obtained with reduced restraint set, display a similar global conformational drift and architecture variation, either the least redundant or randomly picked up restraints are removed. Nevertheless, the removal of the least redundant restraints produces a specific alteration of the β barrel shape and of the packing of hydrophobic residues close to the mutated residue 28.

### 6.3 The instability of nph SH3 mutants is enhanced by using a reduced NMR restraints set

The conformational drift increases for the mutated sequences L28A and L28P with respect to the WT sequence, for the sets **full **(Figure 2a-c) and **reduced **(Figure 2d-f). The increase of conformational drift for mutants is in qualitative agreement with the experimental observations on SH3 stability [9]. The comparison of regression lines, drawn as black lines, proves that similar conformational drifts are observed for a given mutated sequence whatever the set of restraints used. But, the coordinate RMSD gives only a global description of the structural variations.

The mutations mostly increase distances between centers of mass (Figures 3a,b) what-ever the restraint set **full **or **reduced **is used. However, the ranges of distances variations (segments in Figures 3a,b) are enlarged when the set **reduced **is used, and the largest variation ranges (2-5 Å) are observed for the distances (nc/a, n/c, hp/rt, a/rt, src/rt, a/src) involving the N and C terminal regions or the RT loop. As among the 167 restraints involving residues from the RT loop, only two are removed to produce the reduced set of restraints, the motion of the RT-loop apart from the core is not induced by a lack of restraints.

In the mutant proteins, the sidechains of residues neighboring the residue 28 and involved in the SH3 hydrophobic core (Y5, L27, V29, A40, V50 and L55) (Additional file 4) move apart from each other for the set **reduced **(Additional file 4b). On the other hand, for the set **full **(Additional file 4a), the distances between sidechains display various be-haviors. The homogeneous displacement observed for the reduced restraint set (ii) reveals a larger packing disruption around position 28. The inter-residue distances increase from WT to L28A and L28P sequences.

The β barrel shape was analyzed along the β axis (Figure 4b,c) by quantifying the barrel inner cavity (Figure 4d,e). The β barrel length (Figure 4b,c) increases by about 1 Å if the least redundant NMR restraints are removed. Moreover, the β barrel profiles vary more for the simulations based on the set **reduced **(Figure 4e) than for the simulations based on the set **full **(Figure 4d). The removal of the least redundant restraints enhances the profile variations observed for the protein mutants. The profile variability is concentrated around the local maxima and minima described in the previous section, and corresponding to the barrel valves: RT loop, n-src loop, distal hairpin. The β barrel is thus destabilized by the changes of the barrel hoops inducing the disjunction of barrel staves.

The trajectories recorded on the nph SH3 mutants reveal tendencies of the RT-loop to move apart from the structure core. The N and C terminal regions tend to move away from each other, and the packing of the core residues located in the vicinity of position 28 tends to be disrupted. The mutant destabilizing effect is enhanced in trajectories based on the reduced set of NMR restraints.

### 6.4 The variations of the nph SH3 conformation agrees with previous information on SH3 unfolding

The folding of several SH3 domains were studied in the literature by extensive molecular dynamics simulations [4-6,42-48], NMR relaxation measurements [49-51], and mutational analysis [52-55]. A Transition State Ensemble (TSE) [2,7,44,48,56-58] similar for the protein unfolding [4,59] and folding [5,6,43,45] transitions is attained by the drifts of the N and C terminal parts, followed by the opening of the RT loop. The local conformational drifts and the distances between the centers of mass observed here depict conformational features in agreement with the information previously available for the SH3 TSE. The results obtained on nph SH3 will be analyzed in more details with respect to the studies performed by Borreguero et al [45], and Ding et al [44].

Borreguero et al [45] showed that a cluster of contacts between the RT loop and the distal hairpin may be stabilized by the hydrogen bonds E16/M48 and L18/M48. In nph SH3, the mean distances between amide hydrogens and carbonyls of L19 and L49 are smaller than 2.5 Å for the WT and L28A simulations based on the set **full**. On the contrary, the L28P mutation or the use of the set **reduced **disrupt these hydrogen bonds, with corresponding mean distances larger than 2.5 Å.

Ding et al [44] monitored on src SH3 the probability of contacts formation among the conformations observed along protein refolding, at the TSE, before the TSE formation (pre-TSE) and after the TSE formation (post-TSE). The probabilities of corresponding contacts formation in nph SH3, were derived from the mean and the standard deviation values of the Cβ-Cβ distances, a contact being formed if the distance is smaller than 7.5 Å. These probabilities were compared to the probabilities values given in Tables 1 and 2 of Ref. [44], and, for 37 Cβ-Cβ pairs analyzed, the number of contacts post-TS, pre-TS or TSE, displays a two-fold increase in the simulations based on the restraint set **reduced**. The removal of least redundant restraints thus induces the nph SH3 conformation to drift toward the vicinity of the TSE described for src SH3.

### 6.5 SH3 energetic stability and water bridges

**Figure 5:**
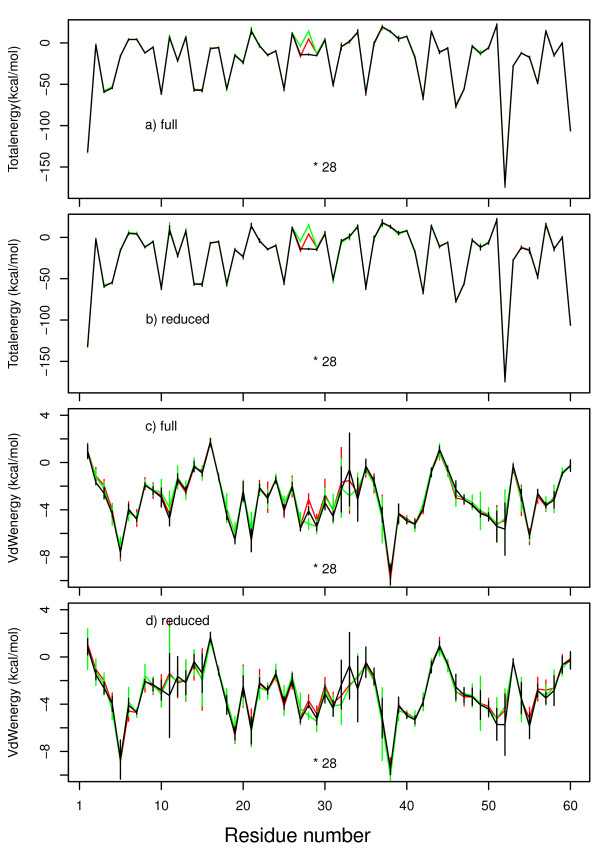
**GBSA energy profiles per residues calculated on the MD trajectories based on the sets **full **(a,c) and **reduced **(b,d) of restraints and recorded on the WT (black), L28A (red) and L28P (green) sequences**. (a,b) total GBSA energy, (c,d) van der Waals energy. The error-bar dimensions have been scaled by a factor 2 in order to put in evidence the differences observed in energy variability. The residue 28 is marked with an asterisk.

The energetic stability of the different nph SH3 sequences were compared by calculating the GBSA profiles on the sequence residues, as described in Materials and Methods. The profiles of total GBSA energies are superimposed whatever is the set of restraints used (Figures 5a and 5b), except for the mutated residue 28 and the residue L27, which display less favorable GBSA energy in the mutants. As the residue L27 is pointing toward the protein interior, its energetic instability supports the distortion of the protein core, in agreement with the previous observations on the β-barrel shape and on the hydrophobic core residues.

The instability of the SH3 core, in the vicinity of the mutation, is furthermore supported by the van der Waals energy profiles in the GBSA energy. Indeed, these profiles show a variability among the 8 runs of each simulation (Figures 5c,d), and this variability is increased for residues located in the vicinity of L28. Other protein residues in the region 7-12 corresponding to a part of the RT loop, and in the region 45-50, corresponding to the last β strand, show also variations of van der Waals energies. An increase of variability and a broader distribution along the sequence are observed if the set **reduced **(Figure 5d) is used: this is in agreement with the increased distortion of the β barrel profile observed for the same restraints set (Figure 4e). Conversely, the observations made on the van der Waals profiles prove that the origin of the β-barrel distortion is effectively the disruption of hydrophobic interactions.

**Figure 6:**
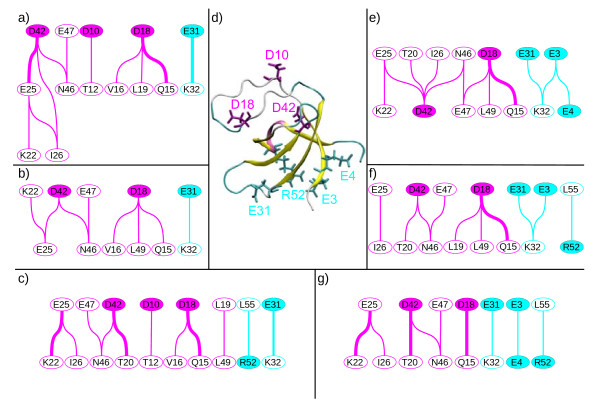
**Water bridges networks observed in simulations based on the sets (a-c) **full **or (e-g) **reduced **of NMR restraints**. The edges materialize the water bridges, formed at least more than 40% of the simulation time for the sequences WT (a,e), L28A (b,f) and L18P (c,g). The vertex stand for the amino acids involved in these networks, the ones with a colored background being the hubs of a sub-network and the others only members. Thicker edges point out bridges formed more than 60% of the MD. (d) The central nph SH3 structure, in new cartoon, exhibits in cyan or in magenta licorice the hub residues involved in water bridges. The colors of the residues correspond to the background color of the vertexes in a-c and e-g.

The MD simulations in explicit solvent make possible the observation of the behavior of water molecules. The water bridges corresponding to water molecules in which two atoms are closer than 2.5 Å of a protein hydrogen bond donor or acceptor group, and present in more than 40% of the MD simulation time, are located around two groups of hub residues (Figure 6d): (i) D10, D18, D42, colored in magenta in the RT loop, and (ii) E3, E4, E31, R52, colored in cyan on the opposite side of the protein, involving residues from the n-src loop, the α helix and the strand β2, and close to the mutated residue L28.

The detected water bridges are plotted as edges of a network relating the interacting protein residues. For the sets **full **(Figure 6a-c) and **reduced **(Figure 6e-g), the most branched network is observed on the WT protein (Figures 6a,e). The less connected network, observed on protein mutants, points out the reduced cooperativity between water molecules, in agreement with the lower stability of the mutants.

## 7 Discussion

The hierarchy of NMR restraints defined by the redundancy was used to produce three sets of starting points for MD simulations, these starting points being NMR conformers calculated with the sets **full**, **reduced **and **random**. All sets of NMR conformations display similar structural and convergence features, and thus equivalent enthalpic properties. The protein fold appears to be stable during the MD runs recorded starting from WT SH3 conformers generated with the full set (i) of restraints. This proves that the force field ff99SB [18] used for MD simulations, is accurate enough to keep stable the structure of the WT nph SH3 domain, in agreement with the NMR experimental observations [9].

The removal of the least redundant NMR restraints (set (ii)) or of randomly chosen restraints (set (iii)) induces similar conformational drifts of the protein. However, a closer analysis of the simulations reveals that the β barrel architecture and the packing of hy-drophobic residues close to the mutated residue L28, are more destabilized if the reduced restraint set is used. The hierarchy of protein contacts, derived from the redundancy of the NMR restraints, is thus related to the funnel [34] shape of free energy around the native state [60].

The mutated residue L28 and its neighbors are involved in the least redundant NMR restraints. The destabilizing effect of the L28 mutations can be thus predicted from the mutual influence of protein topology and NMR restraint geometry, and this could be a general trend of protein unfolding. More physically, the less branched networks of water bridges found for the mutated sequences indicate that the mutations disturb the protein hydration. This hydration instability may arise from the expansion of the β barrel as well as from the distortion of the local hydrophobic interactions, shown in the GBSA profiles, and in the packing defects of hydrophobic residues close to residue 28.

The use of NMR restraint redundancy to destabilize nph SH3 underscores the importance of the protein topology [4] and of the sequence [55]. Indeed, the effect of restraint removal depends mainly on topology, as the WT SH3 displays β barrel instability when the least redundant restraints are removed. On the other hand, mutational effects on the protein stability add to topological effect regardless of whether the full (i) or the reduced (ii) restraint set is used. The removal of the least redundant NMR restraints allows one to enhance protein instability in very short 5 ns MD trajectories, recorded in explicit solvent and using an all-atom force-field.

The work described here is certainly a preliminary one, and the results should be confirmed on other proteins. Nevertheless, the previous use of NMR data-sets to obtain information on thermodynamics of protein stability [36,37] and the relationship of the proposed method with the Gaussian Network Model [38-40], gives strong additional support to the protocol proposed here.

The simplified approaches, as the G*ō *model [61], were widely proved to be essential in the exploration of protein unfolding pathways [62]. The approach proposed here does not allow the exploration of the full protein unfolding pathways, but rather concentrates on the initial steps of this pathway. On the other hand, as the force field used here is not a simplified one, it allows higher precision in the description of the protein instability. In particular, the use of explicit solvent allows one to analyze the role of water molecules in the protein destabilization.

This approach can be applied to any protein structure for which experimental NMR distance restraints are available. Restraint redundancy depends mainly on their distribution in the structure, and is thus indirectly related to the internal protein mobility. The use of the NMR restraint redundancy to explore protein stability is thus similar to sample rare fluctuations in native protein states from the use of NMR hydrogen exchange information [63].

## 8 Conclusions

The instability of WT and mutants of nph SH3 domain was analyzed using MD simulations in explicit solvent. The profile of the β barrel inner cavity was found to be more sensitive to the MD starting points than the global conformational drift.

The present work also permitted to validate a method for destabilizing the conformations of NMR protein structures without biasing the free energy surface. Indeed, the removal of the least redundant NMR restraints [11] allows one to destabilize the protein over short MD trajectories. Besides, the stability difference between WT protein and mutants, is enhanced by the removal of the least redundant restraints, showing that the mutant instability arises from the β barrel shape alteration coupled to an increase of hydrophobic packing defects and to a less connected network of water bridges.

## 9 Abbreviations

MD: molecular dynamics; NMR: Nuclear Magnetic Resonance; WT: wild-type; nph: nephrocystin; NOE: nuclear Overhauser effect

## 10 Authors' contributions

NDS calculated the NMR conformers, performed the molecular dynamics simulations and most of the analyses. LM implemented the analysis of the β-barrel profile, as a constrained smooth optimization problem. MN gave conceptual and technical advice and implemented the structure calculation protocols. TEM designed the study and coordinated the project. TEM, NDS and LM wrote the paper. All authors read and approved the final manuscript.

## Supplementary Material

Additional file 1 **Analysis of the β barrel geometry**. The analyses was conducted in two steps (see Materials and Methods "The β barrel architecture"): (a) determination of the main cylinder axis, (b) determination of local cylinders along the main axis, describing the profile of the β barrel inner cavity. Click here for file

Additional file 2 **Distribution fo the *I*_*uni,r *_information obtained with QUEEN as a function of the restraint index *r***. The *I*_*uni,r *_values of the nine least redundant restraints are colored in red. Click here for file

Additional file 3 **Case study of the pentahedron**. (a) Scheme of the pentahedron defined by all possible distance NMR restraints between the spin nuclei *i*, *j*, *k*, *l *and *m*. The distance restraints are drawn as lines. (b) Variation of the distance restraints in the pentahedron if the restraint between *k *and *j *is removed. Click here for file

Additional file 4 **Mean distances (Å) observed between residues of the hydrophobic core of nph SH3, in the MD trajectories**. They are based on the (a) the full set of NMR restraints of the PDB entry 1S1N, (b) the reduced set of restraints where the least redundant restraints were removed, (c) randomly-reduced sets of restraints, where randomly picked-up restraints were re-moved. The distance values are plotted as bars, colored in black, red and green, for the WT, L28A and L28P sequences. The distances were calculated between sidechain carbons of the residues quoted in abscissa. V50-Cγ2/V29-Cγ1, L55-Cδ1/V29-Cγ2, Y5-Cδ1/V29-Cγ2, A40-Cβ/L27-Cδ2, V29-Cγ2/L27-Cδ2. Click here for file
