# Supplementary figures and images for: The redundancy of NMR restraints can be used to accelerate the unfolding behavior of an SH3 domain during molecular dynamics simulations
Source: BMC Struct Biol. 2011 Nov 24;11:46. doi: 10.1186/1472-6807-11-46 (PMC3274457; doi:10.1186/1472-6807-11-46)

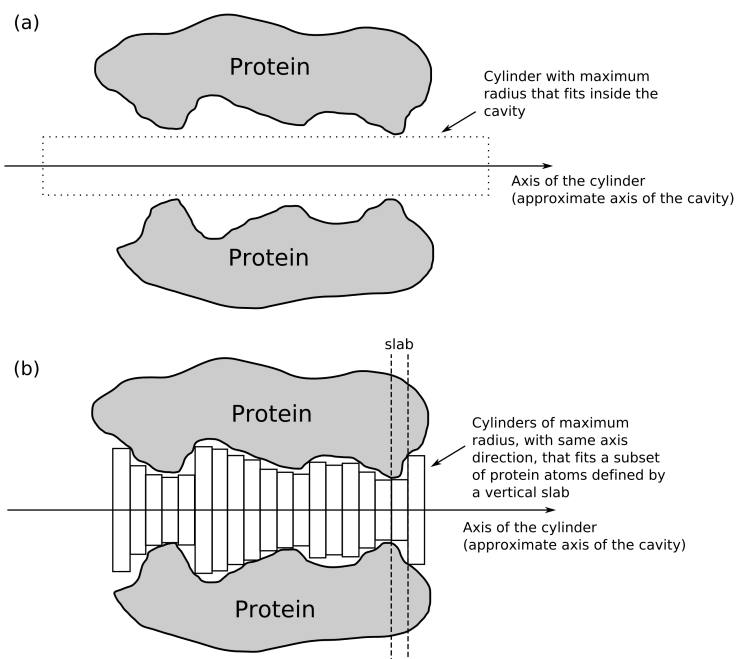

Supplement: Additional file 1 — Analysis of the β barrel geometry. The analyses was conducted in two steps (see Materials and Methods "The β barrel architecture"): (a) determination of the main cylinder axis, (b) determination of local cylinders along the main axis, describing the profile of the β barrel inner cavity. [file 1472-6807-11-46-S1.PDF]

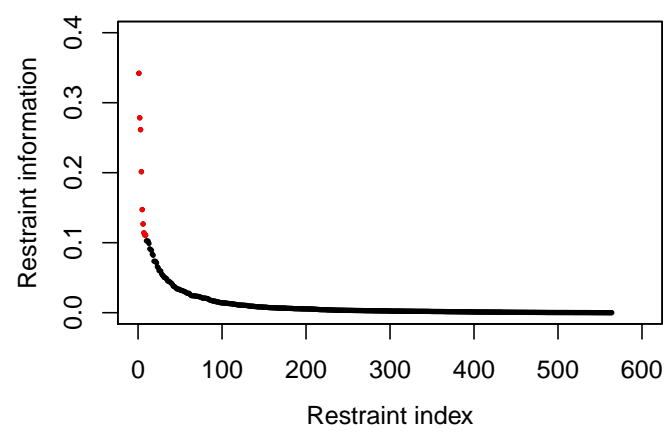

Supplement: Additional file 2 — Distribution fo the Iuni,r information obtained with QUEEN as a function of the restraint index r. The Iuni,r values of the nine least redundant restraints are colored in red. [file 1472-6807-11-46-S2.PDF]

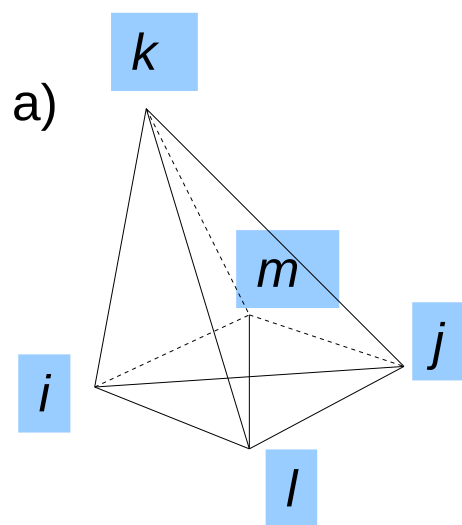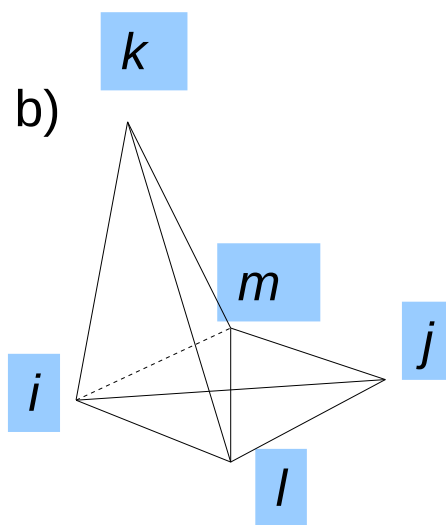

Supplement: Additional file 3 — Case study of the pentahedron. (a) Scheme of the pentahedron defined by all possible distance NMR restraints between the spin nuclei i, j, k, l and m. The distance restraints are drawn as lines. (b) Variation of the distance restraints in the pentahedron if the restraint between k and j is removed. [file 1472-6807-11-46-S3.PDF]

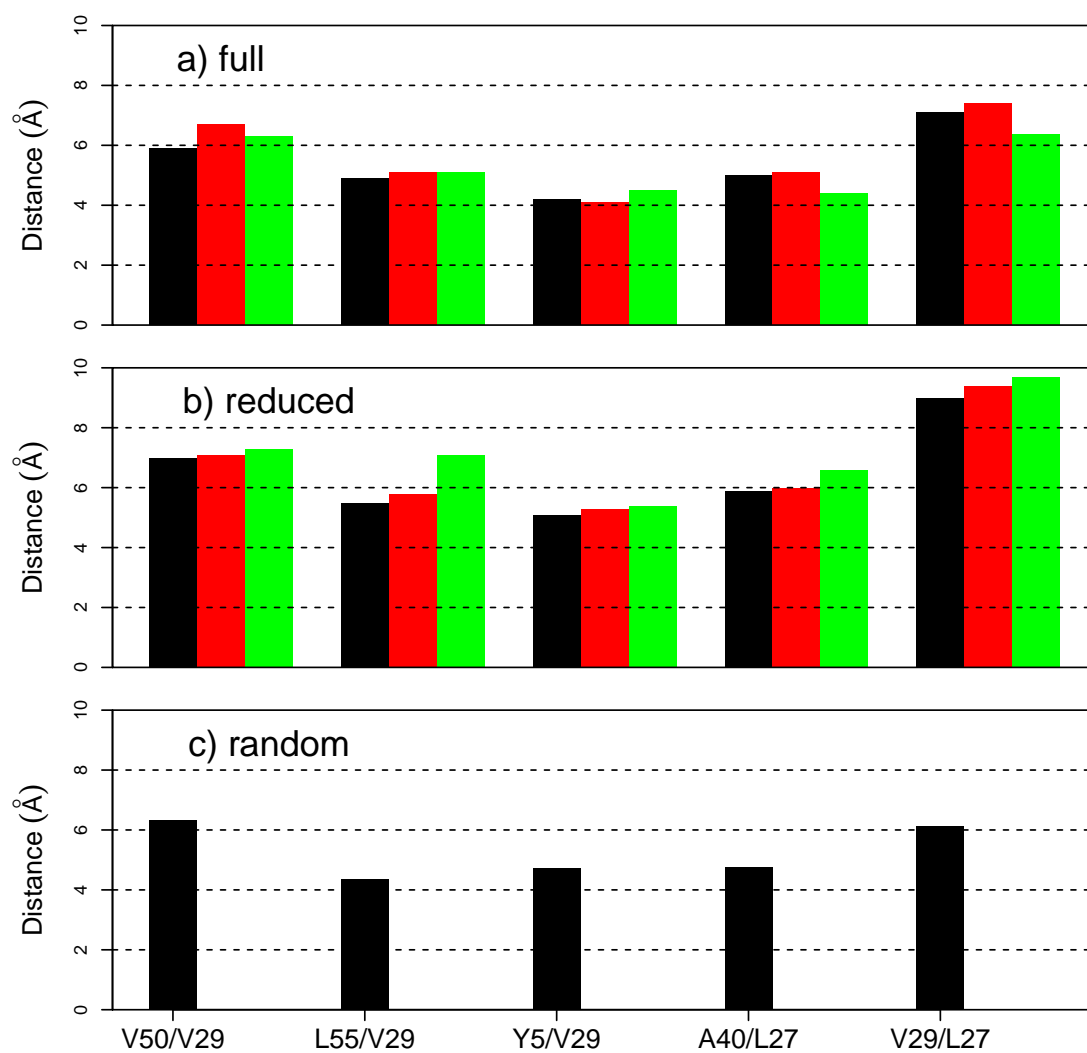

Supplement: Additional file 4 — Mean distances (Å) observed between residues of the hydrophobic core of nph SH3, in the MD trajectories. They are based on the (a) the full set of NMR restraints of the PDB entry 1S1N, (b) the reduced set of restraints where the least redundant restraints were removed, (c) randomly-reduced sets of restraints, where randomly picked-up restraints were re-moved. The distance values are plotted as bars, colored in black, red and green, for the WT, L28A and L28P sequences. The distances were calculated between sidechain carbons of the residues quoted in abscissa. V50-Cγ2/V29-Cγ1, L55-Cδ1/V29-Cγ2, Y5-Cδ1/V29-Cγ2, A40-Cβ/L27-Cδ2, V29-Cγ2/L27-Cδ2. [file 1472-6807-11-46-S4.PDF]
